# Interplay between Short- and Long-Term Plasticity in Cell-Assembly Formation

**DOI:** 10.1371/journal.pone.0101535

**Published:** 2014-07-09

**Authors:** Naoki Hiratani, Tomoki Fukai

**Affiliations:** 1 Department of Complexity Science and Engineering, The University of Tokyo, Kashiwa, Chiba, Japan; 2 Research Fellow of Japan Society for the Promotion of Science, Chiyoda, Tokyo, Japan; 3 Lab for Neural Circuit Theory, RIKEN Brain Science Institute, Wako, Saitama, Japan; 4 Core Research for Evolutional Science and Technology, Japan Science and Technology Agency, Kawaguchi, Saitama, Japan; University Paris 6, France

## Abstract

Various hippocampal and neocortical synapses of mammalian brain show both short-term plasticity and long-term plasticity, which are considered to underlie learning and memory by the brain. According to Hebb’s postulate, synaptic plasticity encodes memory traces of past experiences into cell assemblies in cortical circuits. However, it remains unclear how the various forms of long-term and short-term synaptic plasticity cooperatively create and reorganize such cell assemblies. Here, we investigate the mechanism in which the three forms of synaptic plasticity known in cortical circuits, i.e., spike-timing-dependent plasticity (STDP), short-term depression (STD) and homeostatic plasticity, cooperatively generate, retain and reorganize cell assemblies in a recurrent neuronal network model. We show that multiple cell assemblies generated by external stimuli can survive noisy spontaneous network activity for an adequate range of the strength of STD. Furthermore, our model predicts that a symmetric temporal window of STDP, such as observed in dopaminergic modulations on hippocampal neurons, is crucial for the retention and integration of multiple cell assemblies. These results may have implications for the understanding of cortical memory processes.

## Introduction

Learning and memory are fundamental brain functions supported by hippocampal neural circuits, and long-term potentiation (LTP) and depression (LTD) of synapses are considered to underlie activity-dependent modifications of hippocampal circuits during memory processes. According to the cell-assembly hypothesis [1], [2], memory traces may be represented by functionally grouped assemblies of neurons. Although the mechanism to generate memory traces remains elusive, recent evidence suggests that the groups of neurons activated during behavior are reactivated and reorganized in the awake-quiet and sleep states of animals [3], [4]. These results indicate that memory traces are not static entities driven solely by external stimuli as often assumed in previous theoretical studies, but are actively retained and modulated by spontaneous network dynamics. Moreover, latent modulations, especially selective retention and integration, of memory traces are important in various cognitive tasks [5].

In order to explore the spontaneous modulation of memory traces, we need to model spontaneous activity states with activity-dependent synaptic plasticity, such as spike-timing-dependent plasticity (STDP), in which synaptic weights are modified depending on pre- and post-synaptic spike events occurring in a millisecond-range timescale [6], [7]. Along with long-term plasticity, cortical synapses also undergo short-term plasticity [8], [9]. Short-term plasticity, especially short-term depression (STD), can induce dramatic changes in the characteristic dynamics of recurrent network models such as spontaneous transitions among point attractors [10], [11] or rotational motions in ring attractors [12]. Because STDP depends on spiking activity within a timescale comparable with that of the complex network dynamics, short-term plasticity may significantly influence the processes of cell-assembly formation and retention in recurrent neural networks. In fact, recent experimental results suggest strong influences of short-term synaptic plasticity on memory function [13], [14]. Nevertheless, little is known about interplay between short-term and long-term synaptic plasticity in activity-dependent structuring of recurrent neural networks.

Motivated by the cell-assembly hypothesis [1], [2], here we investigate how STD and STDP may cooperatively generate cell assemblies in response to external stimuli to a recurrent network model also equipped with homeostatic plasticity [15]. We ask whether and how the network retains the memory traces of stimuli for a significantly long period of seconds and minutes in the absence of the stimuli. We explore interactions between multiple cell assemblies during their formation and retention. Our model reveals several conditions on the properties of STD and STDP for the robust maintenance of memory traces in noisy background network activity. In particular, we show that STD plays a crucial role in the retention process. Moreover, we show that modifications of STDP time window, such as observed in hippocampal synapses under dopaminergic modulations [16] or in some neocortical synapses during the development [17], enable the model to dynamically combine multiple cell assemblies into stable clusters with a finite memory capacity.

## Results

**Figure 1:**
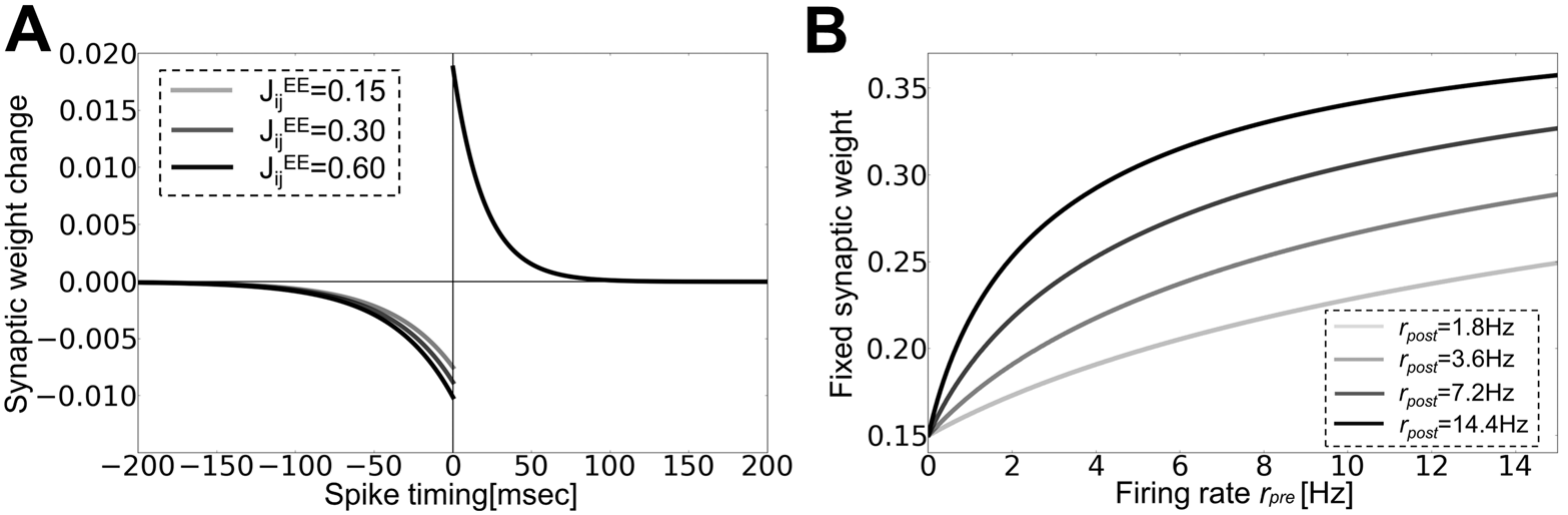
Rate-dependent plasticity through STDP and homeostatic plasticity. (**A**) Spike timing dependence of log-STDP was calculated from equation (7) for given synaptic weights (inset). See Methods for details. (**B**) Firing rate dependence of synaptic weights at the fixed-point of equation (1) representing synaptic dynamics of STDP and homeostatic plasticity. The fixed weights are analytically calculated for various firing rates of pre-neuron *r_pre_* at given firing rates of post-neuron *r_post_*.

We construct a recurrent circuit model consisting of 2500 excitatory neurons and 500 inhibitory neurons that are randomly connected with each other. We introduce short-term plasticity and long-term plasticity into synaptic connections between excitatory neurons, where long-term plasticity is implemented as a combination of log-STDP (Figure 1A) and homeostatic plasticity (Methods). We focus on the effect of short-term depression on the generation and retention of cell assemblies by long-term plasticity.

### Cell assembly formation

If we neglect the effect of synaptic noise, the weight change of synapse *J_ij_^EE^* is approximately written as
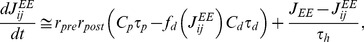
(1)where *r_pre_* and *r_post_* are the firing rates of pre- and post-synaptic neurons, respectively. The first term expresses the effect of STDP, whereas the latter term describes the effect of homeostatic plasticity. When LTP slightly outbalances LTD on average, at its steady state, weights have positive correlations with the firing rates of both pre and post neurons (Figure 1B, for a given *r_post_*) due to relatively strong homeostatic plasticity. If a synaptic weight is large, on average it decreases not only for low input/output rates but also for high firing rates due to the weight dependence of LTD term, so the network tends to be stabilized at a finite firing rate with robustly configured synaptic weights.

**Figure 2:**
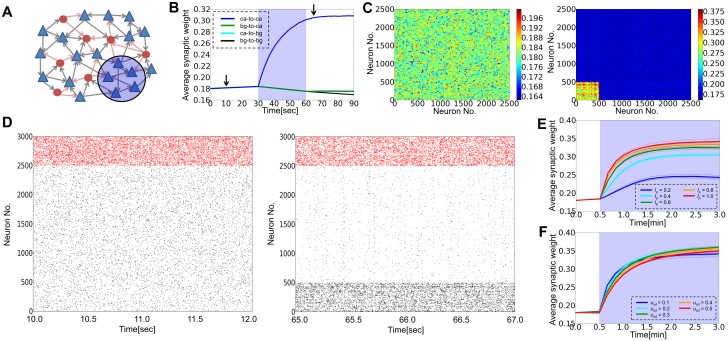
Cell assembly formation by external input for arbitrary strength of STD. In all panels, “ca” stands for a cell assembly and “bg” for background neurons that do not belong to the assembly. The strength of STD was set as *u*_sd_ = 0.1 in simulations from panel B to E. (**A**) Schematic illustration of the model. We stimulate some of excitatory neurons (blue shaded area) in a randomly connected recurrent neural circuit. Triangles indicate excitatory neurons, whereas circles represent inhibitory neurons. (**B**) Time evolution of the average synaptic weights within the selected cell assembly (blue), from background excitatory neurons to the assembly (green), from the assembly to background excitatory neurons (cyan), and outside the cell assembly (black). (**C**) Synaptic weight matrices of excitatory connections are shown before (left) and after (right) the application of external input (arrows in **B**). Excitatory neurons are separated into 100 bins to calculate the average weights. (**D**) Raster plots of spiking activity before (left) and after (right) the application of external input, where red dots represent inhibitory spikes and black dots show excitatory spikes. The temporal position of dots are represents the update timing of the spiking state. Neurons 1 to 500 belong to the cell assembly. (**E**) Dynamics of the average synaptic weight within the cell assembly calculated for various magnitudes of external input *I_p_*. Thin lines are the results from individual simulation trials, and the thick lines are the averages of five simulation trials at each parameter value. (**F**) Dynamics of the average synaptic weight within the cell assembly calculated at *I_p_* = 1.0 for various values of the release probability *u_sd_*.

First, we consider the effect of STD on cell assembly formation by selectively stimulating an excitatory neuron group (Figure 2A). The weights of synaptic connections are initially random (Figure 2C left), and the network shows an irregular spontaneous activity state with low firing rates (*r_E_* = 1.5–2.0Hz, *r_I_* = 10–15Hz) (Figure 2D left). Then, we apply a constant external current *I_p_ = 1.0* to randomly chosen 20% of excitatory neurons for 30 seconds. During this external stimulation, those 20% of excitatory neurons constantly fire at a high firing rate of 10–15Hz, and as a result synaptic connections among these neurons become strong (Figure 2B, blue shadow indicates the neurons receiving the external stimulus) due to long-term potentiation caused by the high firing rates of presynaptic and postsynaptic neurons (as shown in Figure 1B). After the stimulus is turned off, the average connection strength between stimulated neurons is significantly larger than other excitatory connections (Figure 2C right), and the firing rates of these neurons are also higher than others (Figure 2D). Thus, a cell assembly can be formed in a stimulus-dependent manner. The average weight of synapses belonging to the assembly becomes larger for stronger input current (Figure 2E). The observed phenomena are qualitatively the same for simulations at different values of the release probability parameters (Figure 2F), implying that the details of STD are not essential for the generation of cell assemblies.

### Cell assembly retention

**Figure 3:**
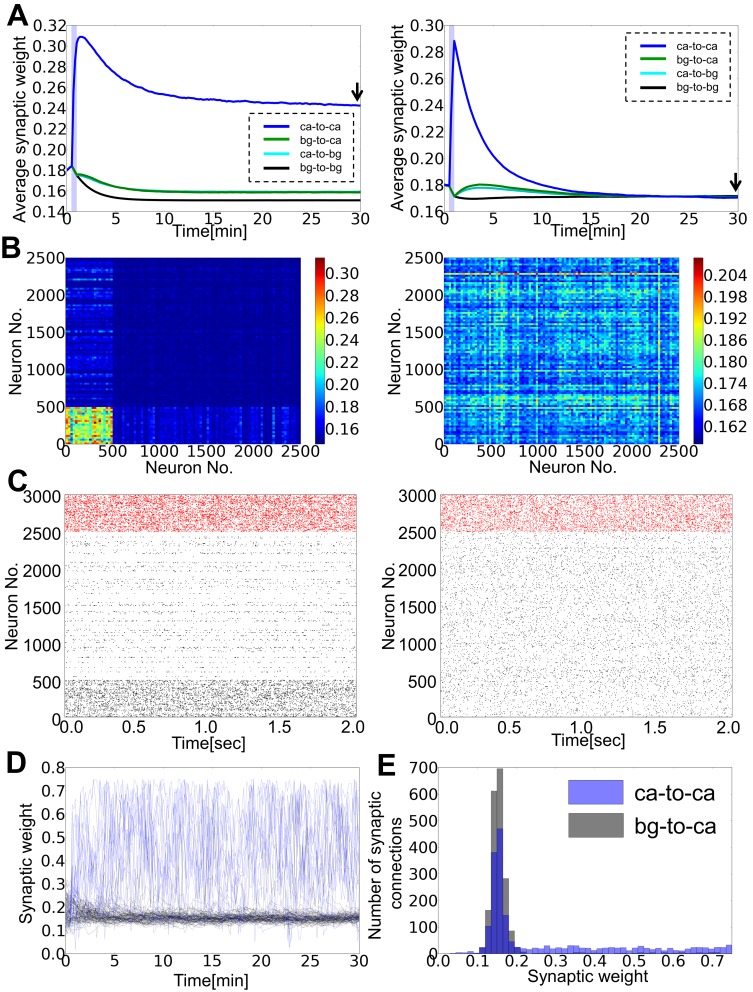
Strong STD disturbs cell assembly retention. (**A**) Time evolution of average synaptic weights within the selected cell assembly (blue), from background excitatory neurons to the assembly (green), from the assembly to background neurons (cyan), and between background excitatory neurons (black). The left and right panel show results for *u_sd_* = 0.1 and *u_sd_* = 0.5, respectively. (**B**) Weight matrices of excitatory synaptic connections calculated at *t* = 30 min are shown for *u_sd_* = 0.1(left) and *u_sd_* = 0.5(right). (**C**) Raster plots are displayed for the weight matrices shown in **B**. (**D**) Dynamics of individual synaptic weights is shown on one excitatory neuron in the assembly. Blue lines correspond to weights from neurons belonging to the assembly, whereas gray lines to those from background excitatory neurons. (**E**) Distributions of input synaptic weights were calculated from simulation data at *t* = 26.7–30 min for the neuron shown in **D**.

Because neurons belonging to a cell assembly interact with neurons outside it, the stability of cell assembles in the absence of external stimuli is not trivially ensured. In fact, this stability crucially relies on the properties of STD, as shown below. After the termination of stimuli, the average synaptic weights in general return slowly toward the initial values, although they eventually converge to certain values that may not coincide with the initial ones. When the release probability is small (*u_sd_* = 0.1), the weights inside the cell assembly is distinctly larger than other weights (Figure 3A left), and the trace of the cell assembly remains visible even after 30 minutes in both synaptic weights (Figure 3B left) and neural activity (Figure 3C left). Synaptic weights between neurons inside the cell assembly and background cells (i.e., cells not belonging to the assembly) are somewhat larger than those among background cells, as the high rate of presynaptic (postsynaptic) firing enhances synaptic weights due to the firing-rate dependency of STDP. Background neurons also change their firing pattern because the balance condition of the network changes after learning. On each excitatory neuron belonging to the cell assembly, synaptic weights from other cells in the assembly remain large showing large fluctuations, whereas the weights from background cells stay small (Figure 3D). Eventually, the synaptic weights on assembly cells obey a long tailed distribution in which the long-tail mainly consists of synapses from other neurons in the assembly, while that of background neurons constitutes a more Gaussian-like distribution (Figure 3E). In contrast, for strong STD (*u_sd_* = 0.5), spontaneous activity gradually erases the cell assembly (Figure 3A right), and both neural activity and the synaptic weight matrix become nearly random after several minutes (Figure 3B right, Figure 3C right). These results indicate that STD is highly influential on the cell assembly retention: especially strong STD disturbs the retention.

**Figure 4:**
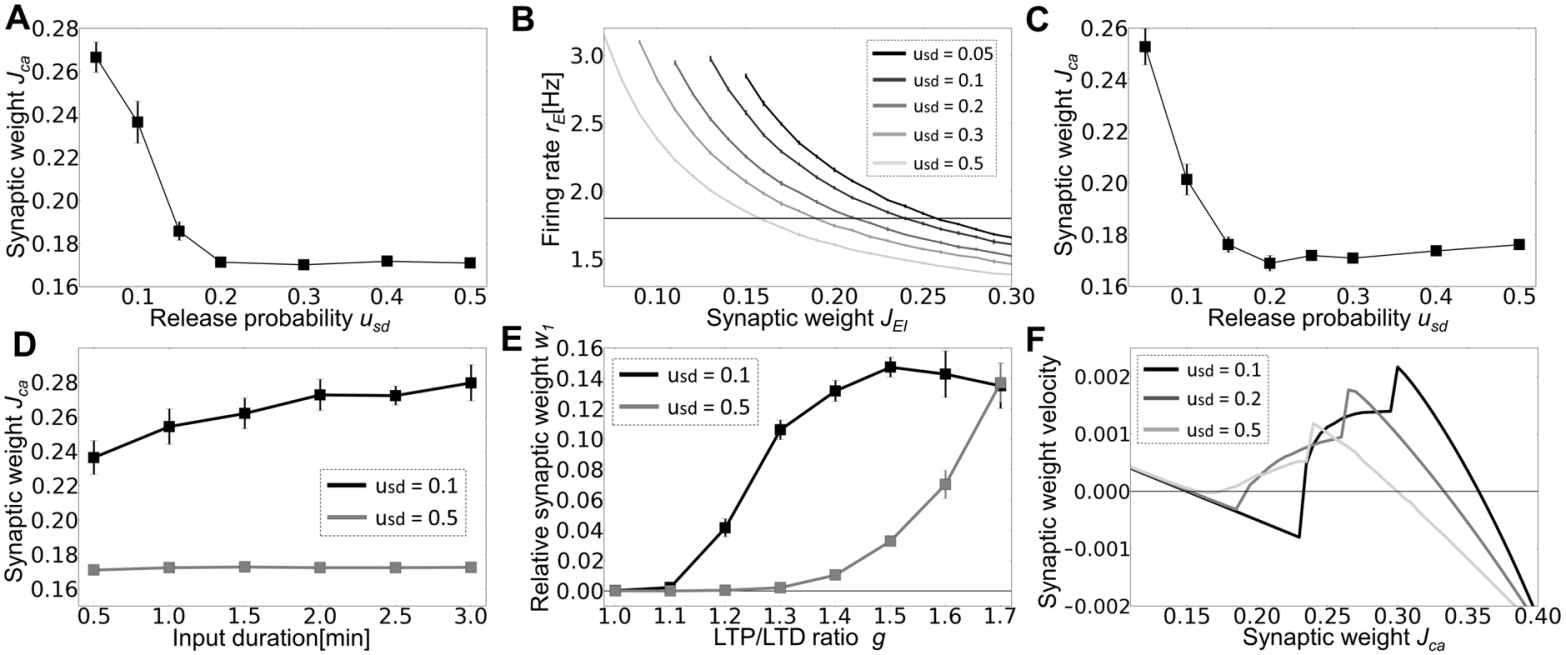
Crucial effects of STD on cell assembly retention. Unless otherwise mentioned, error bars represent the standard deviation obtained by five simulation trials. The results shown in panel **A** and **C** to **E** were calculated at *t* = 30 min. (**A**) Relationship between the release probability *u_sd_* and the average synaptic weight within the cell assembly. The results were averaged over five simulation trials. The weights of synapses other than *J*_EE_ were constant. (**B**) Relationship between inhibitory-to-excitatory synaptic weights *J_EI_* and the average firing rates of excitatory neurons is shown in a network model without long-term synaptic plasticity. Horizontal line indicates *r_E_* = 1.8 Hz. (**C**) Release probability dependence of the average synaptic weight within the assembly is shown. Each plot was calculated using the value of *J_EI_* which sets the average firing rate of excitatory neurons to 1.8 Hz. (**D**) Relationship between the average synaptic weight within the assembly and input duration is shown. (**E**) The dependence of the relative synaptic weight *w_1_* to LTP/LTD ratio *g* = *C_p_τ_p_*/(*C_d_τ_d_*), which we varied by changing the value of *C_p_* between 0.015 and 0.0255. (**F**) Mean-field approximation gives the velocity of weight change as a function of the synaptic weight. Each line is calculated from equation (10) using the steepest descent method from various initial conditions.

Figure 4A shows the average synaptic weight inside the cell assembly observed after 30 minutes. The value decreases monotonically as the release probability increases. When the release probability is larger than 0.2, the assembly becomes indistinguishable from other synaptic weights. We studied whether the above results are a direct consequence of STD or merely reflect the effect of other parameters modulated by STD. We first checked the effect of inhibitory inputs. When STD is strong, each excitatory neuron generate fewer spikes for the same inputs, thus the excitatory-inhibitory balance of the recurrent network shifts to an inhibition-dominant regime. We calculated the average firing rate of excitatory neurons for various inhibitory connection weights *J_EI_* and release probabilities *u_sd_* at a fixed value of *J_EE_* (Figure 4B). Then, we adjusted the values of *J_EI_* such that excitatory neurons fire at a similar average firing rate (of 1.8Hz) for simulations at different release probabilities, and calculated the average synaptic weight in the cell assembly after 30 minutes of exposure to long-term synaptic plasticity. If the weight dependence on *u_sd_* arises from differences in the excitation-inhibition balance in Figure 4A, the weights would not change their values in these simulations. However, the average weight almost monotonically decreases as the release probability increases (Figure 4C), indicating that inhibitory inputs are unlikely to cause the decrease of synaptic weights.

Next, we considered the effect of input duration. For *u_sd_* = 0.1, longer input duration resulted in slightly larger synaptic weights in the cell assembly. In contrast, the weights were not retained for *u_sd_* = 0.5 even when the input duration was as long as three minutes (Figure 4D). Therefore, a robust retention of cell assemblies is possible only if STD is sufficiently weak. If LTP is sufficiently strong compared to LTD (*C_p_τ_p_*/(*C_d_τ_d_*) > 1.6) cell assemblies also remain stable for large *u_sd_* (Figure 4E). However, such a strong LTP is highly unlikely for cortical synapses. Here, we defined the relative weight *w1* as 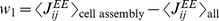 to evaluate the robustness of cell assemblies.

Finally, we numerically solved equation (10) to study the effect of STD on the stability of cell assemblies. We calculated the fixed points of equation (10) for given value of *J_ca_*, and then calculated the weight velocity shown in equation (1) at various values of *J_ca_*. We found that for given release probability *u_sd_*, the numerical solution typically has two stable points corresponding to a state (with small *J_ca_*) in which background neurons are most active and a state (with large *J_ca_*) in which neurons belonging to a cell assembly are almost exclusively active (Figure 4F). As the release probability is increased, the stable fixed point with large *J_ca_* moves to the left side, while the stable point with small *J_ca_* eventually disappears in the analytic treatment. In numerical simulations of the network model, however, the two states become closer and less distinguishable (data not shown), implying that they should merge together at a critical value of *u_sd_* in Figure 4F. This discrepancy around a critical point is considered to arise from the approximations we employed for making the neural dynamics and weight dynamics analytically tractable. For example, we used mean synaptic weights in analyzing neural and synaptic dynamics although the weight distribution is far from a Gaussian (Figure 3E). These approximations presumably oversimplify the dynamics of our network model with highly heterogeneous synaptic weights.

### Interferences between cell assemblies

**Figure 5:**
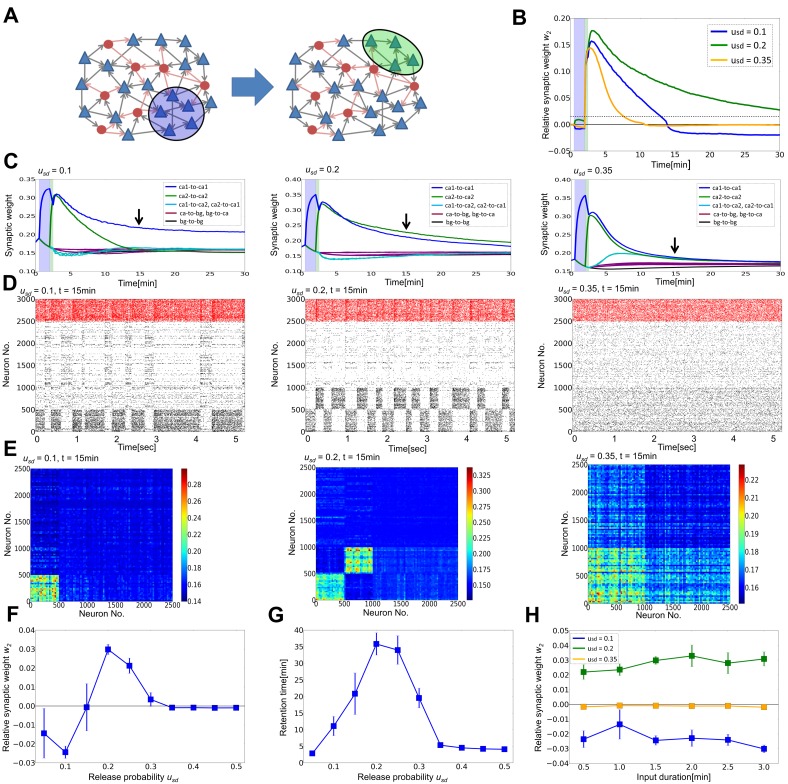
Retention of cell assemblies by weak STD. (**A**) A first external input activates 20% of excitatory neurons (ca1, blue shaded area), and then a second input successively activates other 20% of excitatory neurons (ca2, green area). Neurons not stimulated by the external inputs are regarded as background (bg). (**B**) Time evolution of relative synaptic weight *w_2_*. Blue shade indicates the interval of the first stimulus, and the green shade denotes the second one. We defined the retention time of a cell assembly as the time at which *w_2_* crosses threshold from above (*w_2_* = 0.015: dotted line). (**C**) Time evolution of the average synaptic weight for three values of *u*_sd_. The weights were separately averaged over synapses within and between different cell assemblies and background neurons. In the left and middle panels, black lines for bg-to-bg connections are hidden behind purple lines. (**D**) Raster plots of spiking activity corresponding to the three cases shown in **C**. Color codes are the same as in Figure 2**C**. First 500 neurons belong to the first assembly and the second 500 neurons to the second assembly. (**E**) Synaptic weight matrices of excitatory connections are shown for the above three cases. (**F**), (**G**) The relative synaptic weight *w_2_* and the retention time of ca2 are shown as functions of the release probability *u_sd_*. (**H**) Relationship between the input duration to ca1 and the relative synaptic weight *w_2_* at *t* = 30 min.

The results shown in the previous section have revealed that STD has strong influences on the retention of a cell assembly, but not much on its formation. To further demonstrate the effects of STD on the formation and retention of multiple cell assemblies, we stimulated a randomly chosen 20% of excitatory neurons in a recurrent network that initially had random synaptic weights. Directly after the first stimulation, we stimulated another 20% of excitatory neurons that do not overlap with the first group (Figure 5A). We applied the first stimulus for 90 seconds and the second stimulus for 30 seconds because the application of the second one rapidly weakened recurrent synapses in the first neuron group. During the second stimulus, inhibitory neurons suppress the activity of the first neuron group, and then homeostatic plasticity weakens synaptic connections between these inactive neurons. Under these conditions, the external stimuli generated two cell assemblies in the recurrent network. Here, we ask whether these cell assemblies survive separately, disappear or merge with one another when they undergo spontaneous network activity.

To quantify the different wiring patterns emergent in the network, we define the relative synaptic weight *w*_2_ as
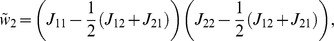


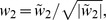
where *J_µν_* is the average weight of synaptic connections from cell assembly *ν* to cell assembly *µ*. The relative weight is normalized such that it has the dimension of synaptic weights. If the two assemblies survive independently, *J_11_* and *J_22_* should be much larger than *J_12_* and *J_21_*, making *w*_2_ strongly positive. On the contrary, if the first assembly survives and the second one disappears, *w*_2_ may take a negative value. If the two assemblies merge into one or both of them disappear, *w*_2_ will be close to zero.

Depending on the value of the release probability, the relative weight acquires positive, negative or almost vanishing values when the network undergoes spontaneous activity (Figure 5B). For small release probability (*u_sd_* = 0.1) both assemblies exhibit high firing rates after the two stimuli, but only one of them remains active after several minutes (Figure 5D, left). Accordingly, the synaptic weight matrix retains memory traces only for the surviving assembly, but not for the other (Figure 5C and 5E, left). Interestingly, the transient state of cell assemblies can show slow oscillations at 0.5–2 Hz (Figure 5D, left), unlike in the previous case with a single cell assembly. If STD is slightly stronger (*u_sd_* = 0.2), the two assemblies are kept activated alternately even 15 minutes (biological time) after the termination of external stimuli (Figure 5D, middle), and the synaptic weight matrix indicates clearly distinct memory traces of these assemblies (Figure 5E, middle). However, we note that these assemblies are not permanently stable and eventually disappear, typically after 30 to 60 minutes (Figure 5E, middle). If STD is further strengthened (*u_sd_* = 0.35), the average synaptic weights rapidly decrease in both assemblies (Figure 5C, E, right) and connections become stronger between the assemblies. As a result, they merge into a large assembly (Figure 5D, right) though this assembly is also unstable and eventually disappears (Figure 5C right).

The relative weight *w_2_* at 30 minutes takes negative values for weak STD (*u_sd_* < 0.15), positive values for intermediate strength of STD (0.15 < *u_sd_* < 0.35), and vanishes for stronger STD (Figure 5F). If we define the lifetime of assemblies as the time at which *w_2_* becomes smaller than 0.1*J_EE_*, the lifetime is maximized when STD is modestly strong (Figure 5G). Therefore, adequately strong STD is necessary for a prolonged retention of stimulus-induced cell assemblies. Varying the duration of the first stimulus does not essentially change these results (Figure 5H), suggesting that the internal dynamics of synapses and neurons determines the lifetime of cell assembles. At *u_sd_* = 0.1, the winning assembly changes from the second to the first if the duration of the first stimulus is about 1–1.5 minutes (data not shown). We also performed simulation with Poisson neuron model to ensure the universality of the results (Text S1 and Figure S1).

### Stability analysis for cell assemblies

**Figure 6:**
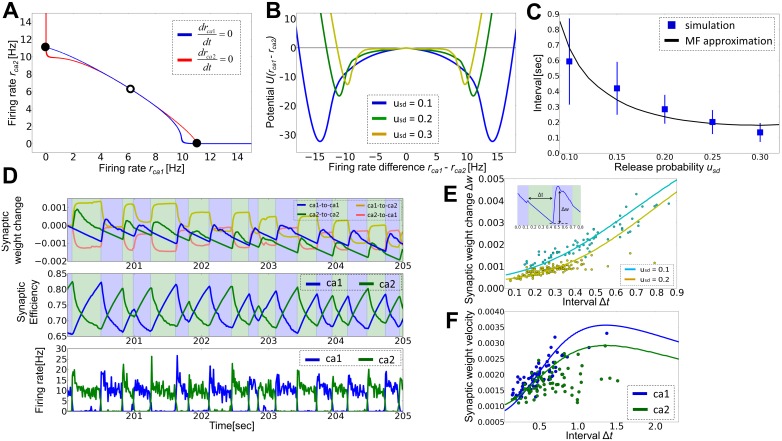
STD induces alternate excitations of assemblies, which enlarges synaptic weights within the assemblies. (**A**) Null-clines of firing rates for a synaptic weight matrix calculated from equation (9). (**B**) Potential function *U* is calculated for the difference in firing rate between two assemblies. The normalization factor *U*_0_ is determined to ensure *U*(0) = 0. (**C**) A monotonic relationship between the release probability and the average interval of the alternation of cell assemblies. The interval was defined as a duration in which one assembly continuously shows higher firing rates than the other. Firing rates were calculated in 10 milliseconds-long time bins. Error bars are the standard derivation of intervals observed during 80 seconds after the stimulus termination in a simulation trial. (**D**), Typical behavior of the average synaptic weights (above), synaptic efficiency for STD (middle), and neuronal firing rates (below). The first (blue) and second (green) cell assemblies show high firing rates alternately. (**E**) Relationship between the interval and synaptic weight change for *u_sd_* = 0.1 (cyan) and *u_sd_* = 0.2 (yellow). Inset illustrates the two quantities shown. The ordinate shows synaptic weight change *Δw* in an interval (*Δt_w_* = 80 milliseconds) starting from the activation of the corresponding cell assembly. Dots are data points obtained from simulation, while solid curves indicate analytic results. (**F**) Interval dependence of the synaptic weight velocity is shown, which was defined as an expected synaptic weight change in a second. Solid curves show the analytic results calculated at *J_ca1_* = 0.311, *J_ca2_* = 0.287, *J_bg_* = 0.156, *r_ca1_* = 13.38 Hz and *r_ca2_* = 12.82 Hz.

We investigate the stability conditions for dual cell assemblies. Because the synaptic weight matrix changes much more slowly than the membrane potentials, we first study the dynamics of average firing rates for a given weight configuration by the mean-field approximation. We derived the null-clines 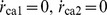 of firing rates by numerically solving equation (9) for a network containing two cell assemblies, that is for a synaptic weight matrix given as: *J_ca1_* = *J_ca2_* = 0.3, and all other excitatory weights as 0.17. The intersections of the two null-clines correspond to the fixed points of the network dynamics. In general, the network has an unstable fixed point and two stable fixed points in which one of the two assemblies displays a non-vanishing firing rate (Figure 6A). Making an approximation that a smaller variable between *r*_ca1_ and *r*_ca2_ is slaved to a bigger one, we obtain the potential function
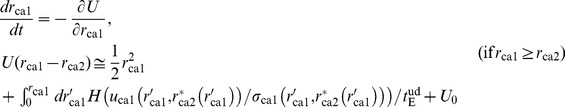
(2)


We should exchange the indices “ca1” and “ca2” when *r_ca1_*<*r_ca2_*. Note that in general we cannot derive a one-dimensional potential function for a dynamical system of more than two variables without such an approximation. We adjust the constant term *U_0_* such that *U(0) = 0* for different values of the release probability.

For a given synaptic weight matrix, the potential barrier separating the two stable states becomes lower as the release probability gets larger (Figure 6B). Driven by random noise, therefore the network state tends to oscillate between the two stable points, each corresponding to one active cell assembly, more frequently for larger release probability. We have already observed this alternation between active cell assemblies in the previous simulations. We confirmed this result by numerical calculations of the average periods of these oscillations following the stimulus termination and a regression analysis with function 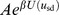
(*A* = 0.0679, β = 0.0691), where *U*(*u*_sd_) is the potential calculated at *u = u*_sd_ (Figure 6C). We note that the average interval is shorter when the amplitude of noise is larger, which typically occurs when the average firing rate of excitatory neurons is high.

We next consider how the evolution of firing rate controls the dynamics of synaptic weights. Synaptic weights within a cell assembly rapidly increase when the assembly is active, and gradually decrease otherwise (Figure 6D above). Correspondingly, the synaptic efficiencies for STD drop sharply at the beginning of the active epoch, and they recover slowly in the silent epoch (Figure 6D middle). In contrast, synaptic weights between the two assemblies undergo significant changes only when a postsynaptic assembly is transiently active (Figure 6D above). To analyze how STD influences this active maintenance of synaptic weights, we investigate the relationship between the interval of cell-assembly activation (i.e. the duration of the silent epoch), *Δt*, and the change in intra-assembly synaptic weights at the beginning of an active epoch, *ΔJ*. The two quantities are positively correlated (dots in Figure 6E), and *ΔJ* tends to be larger for weaker STD (i.e., smaller *u*_sd_), as explained analytically below. When a cell assembly is active, the efficiency of synapses decreases in the assembly until it reaches the equilibrium value 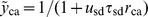. In contrast, during the silent period of an assembly, the efficiencies gradually recover toward an initial level,
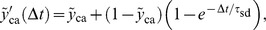
which depends nonlinearly on the value of *u*_sd_. After the silent epoch of length *Δt*, the average firing rate *r’_ca_(Δt)* of the assembly becomes higher than the average firing rate *r_ca_* in the equilibrium state, because the synaptic efficiency 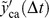 is larger than the equilibrium efficiency 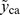. We can calculate the firing rate *r’_ca_(Δt)* by substituting 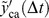 into *y_ca_* in equation (9) (Method). From equation (1), we can then calculate the average weight increase *ΔJ(Δt)* between the neurons in the initial *Δt_w_* milliseconds of the active epoch as



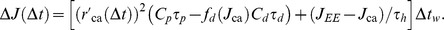



This function calculated from the numerical data observed in simulations (*J_ca1_* = 0.311, *J_ca2_* = 0.287, *J_bg_* = 0.156, *r_ca1_* = 13.38 Hz for *u*_sd_ = 0.1; *J_ca1_* = 0.317, *J_ca2_* = 0.309, *J_bg_* = 0.155, *r_ca1_* = 10.14 Hz for *u*_sd_ = 0.2) fits the actual values well (Figure 6E, solid lines).

We found that the firing rate *r’_ca_(Δt)* generally increases with *Δt*. However, this does not imply that longer *Δt*, which typically occurs for weaker STD, is advantageous for the retention of cell assemblies because the velocity of weight change per unit time, 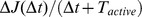, where *T_active_* is the average interval of an active epoch, does not increase monotonically with *Δt*. In Figure 6F, we show the weight velocity calculated by using the average intervals obtained numerically (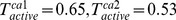 for *u*_sd_ = 0.1). Thus, although longer intervals generate larger weight changes, they also generate more robust stable states of the potential function (Figure 6B), and the alternate activation of two cell assemblies becomes more difficult (see Figure 5D). In contrast, if the strength of STD is in an appropriate range, the two assemblies are alternately activated by noise, enabling the synaptic weights in a resting assembly to increase during its following active period. Although a rigorous analysis of the stability of cell assemblies at relatively strong STD is difficult, we can provide intuition for the observed effects. If STD is weak, an active assembly has a relatively long lifetime. In this case, active assemblies switch only infrequently and the alternate activation can be stable. In contrast, if STD is strong and an active assembly has a short lifetime, active cell assemblies switch frequently and synaptic connections are reciprocally strengthened between the two assemblies, implying that they eventually merge together.

### Crucial effects of STDP time window on the stability of cell assemblies

The results shown in the preceding section reveal that cell assemblies are metastable and can survive synaptic bombardment in spontaneous activity only for a few tens of minutes. Although the storage of episodic memory can be as long as hours and days, biological processes responsible for this are considered to involve cellular and molecular mechanisms [21]. Our results demonstrate how cell assemblies may be maintained against noise through a network mechanism for minutes to hours. The lifetime of assemblies observed in the previous section is much longer than the characteristic time scales of synaptic and neuronal dynamics. However, the lifetime may not be long enough to induce molecular and cellular processes to stabilize patented synapses. Especially, as we will see later, cell assemblies are less stable when more metastable states exist in the network. In this section, we explore a possible solution to this problem.

As in the previous section, we define the relative weight *w_p_* as
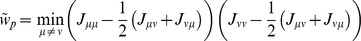


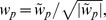
for general cases with more than two cell assemblies, where *J_µν_* is the average synaptic weight from cell assembly *µ* to *ν*. Because it is time-consuming to train the network with many cell assemblies, hereafter we construct a synaptic weight matrix by hand such that it contains *p* assemblies each consisting of *N_E_a* excitatory neurons (Methods). We examine what STDP rule may retain stable cell assemblies.

**Figure 7:**
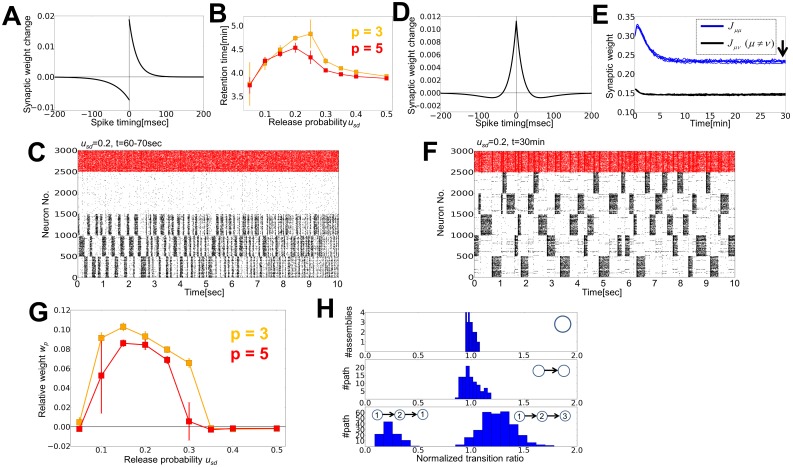
The retention of cell assemblies with Hebbian and symmetric STDP windows. (**A**) An asymmetric STDP window was calculated for *J_ij_^EE^* = 0.15. (**B**) The retention time significantly varies with the release probability of STD. We defined the retention time as a period with a sufficiently large relative weights: *w_p_*>0.1*J_EE_*. (**C**) Raster plot of spiking activity is shown for the Hebbian STDP rule shown in **A**. (**D**) A symmetric STDP window was calculated for *J_ij_^EE^* = 0.15. (**E**) Dynamics of the average synaptic weights at *u_sd_* = 0.2 within (blue) and between (black) assemblies. (**F**) Raster plot of spiking activity for the symmetric STDP rule shown in **D**. (**G**) Relationship between the release probability *u_sd_* and relative weight *w_p_* at *t* = 30 min. (**H**) (top) We constructed a histogram of the number of activation over all cell assemblies shown in **F**. The abscissa shows the number of activation of each assembly normalized by the average number of activation of all assemblies. (middle) We calculated a histogram for the occurrence of all possible 20 (5×4) sequential transitions between two assemblies. The occurrence number of each transition was normalized by the average occurrence number over all transitions. (bottom) Histograms of triplet transitions, such as assembly 1 → 2 → 1 (left) and 1 → 2 → 3 (right), are shown after a normalization by all possible 80 (5×4+5×4×3) triplet transition patterns. All three histograms are obtained from the results of five simulation trials.

We first investigate models with a relatively small number of assemblies (*p* = 3 or 5). When STDP is asymmetric-Hebbian and *u*_sd_ has an adequate value (Figure 7A, B), the cell assemblies are activated independently and randomly for a while. However, the transient network state switches between different activation patterns of cell assemblies until it displays a sequential activation pattern of assemblies, which in turn evolves into synfire-like activity (Figure 7C, at t = 60–70 sec). However, this activity is unstable and does not persist. Thus, the network eventually returns to random firing states. The lifetime of cell assemblies is longest at a moderate release probability (Figure 7B). We found that such a transient state evolution is typical for the asymmetric STDP window.

Cortical synapses are known to change their STDP rules [18], [19]. In particular, under the presence of dopamine, the STDP window of glutamate synapses turns nearly symmetric in rat hippocampus [16]. Moreover, during the developmental stage, excitatory connections from layer 4 to layer 2/3 display symmetric STDP [17]. So, we investigated whether a symmetric window function may change the stability of cell assemblies with the following STDP window (Figure 7D):
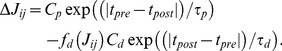
(3)


We performed numerical simulations of this network for *p* = 3 or 5 and *u*_sd_ = 0.2. The average weights within cell assemblies converge to stable values after several minutes (Figure 7E). The network persistently and irregularly activates all cell assemblies one by one, and this state remains stable even after 30 minutes (Figure 7F). Consistent with our previous results, such irregular stable states appear only when the strength of STD is in an adequate range (Figure 7G). We next examined whether the activation pattern is random or biased by analyzing spike data taken from 10 to 30 minutes after the initiation of spontaneous activity. We found that all assemblies are activated for nearly the same amount of time (Figure 7H, top). The frequencies of sequential transitions between two assemblies show no statistically significant bias (Figure 7H, middle). In contrast, sequences involving the reactivation of an assembly, such as 1→2→1, are less likely to occur because STD of mutual excitation in an active assembly suppresses the immediate reactivation of the same assembly. Therefore, the frequencies show some bias among triplets of assemblies (Figure 7H, bottom). The occurrence of monotonous short sequences of cell assemblies is a typical problem in recurrent networks with STDP [20]. It is noteworthy that excitatory weight matrices do not develop short sequences in the present model because synaptic efficiency does not recover in a short time.

**Figure 8:**
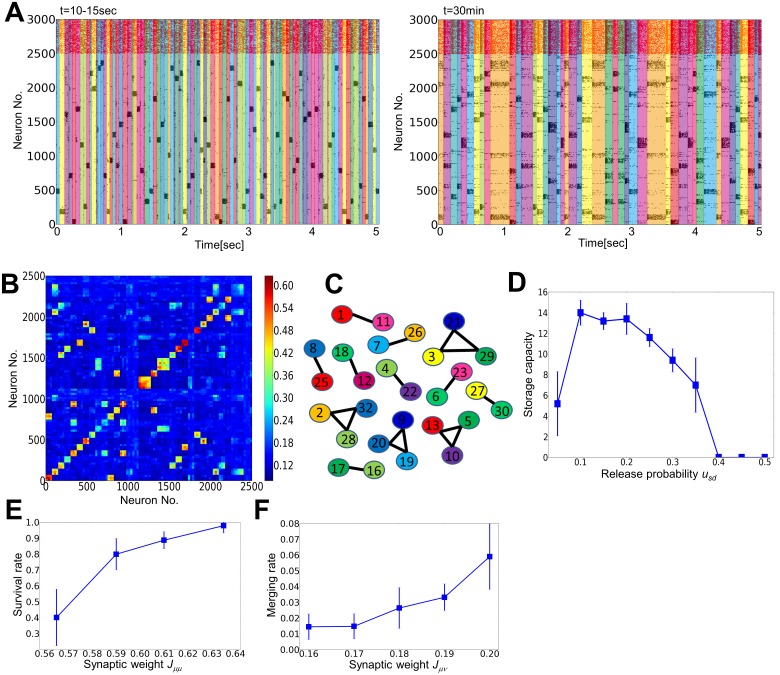
Merging and oblivion of cell assemblies through spontaneous activity. (**A**) Raster plot of spiking activity in a network embedding 32 cell assemblies. Active epochs of initial assemblies are shown by different colors in the left panel, while those of merged assemblies are shown in the right panel. (**B**) Synaptic weight matrix after 30 minutes of spontaneous activity. (**C**) A graphical representation of the merged connection matrix, where each numbered circle corresponds to an initial assembly. (**D**) Relationship between the storage capacity and the release probability. (**E**) The survival rate of each assembly depends on the initial magnitudes of intra-assembly synaptic weights. We separated cell assemblies into four groups according to the initial weight values (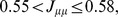 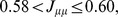 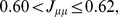 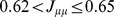: the boundaries were decided such that each group contains 5 to 15 assemblies) and calculated the fraction of the assemblies that survived in the reorganization. See Methods for other details of the simulations. (**F**) The rate of merging of a cell assembly as a function of the initial synaptic weight. As in **E**, we separated 992 inter-assembly connections into five groups (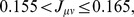 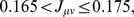 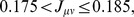 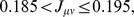 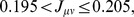) so that each group contains more than 100 assemblies.

Does the retention of cell assemblies sustained by random activation shown above in neural networks with small numbers of assemblies hold for large-scale network models? To answer this, we performed simulations of a network containing a large number of cell assemblies. We set model parameters as *u*_sd_ = 0.2, *p* = 32, *a* = 0.03, *J*_ca_ = 0.7, and *J*_bg_ = 0.15. Note that the size of this network is the same as the previous ones, but each cell assembly now consists of 75 neurons while 500 in previous models. The network initially retains all assemblies by randomly visiting them (Figure 8A, left). After 30 minutes passed, however, some cell assemblies survived stably, but others simply disappeared or merged into bigger stable assemblies (Figure 8A, right). Activity-dependent reorganization of synaptic weight matrix *J_µν_* underlies these changes in the spontaneous activity pattern (Figure 8B). We may define “the storage capacity” of the recurrent network as the number of independent assemblies surviving the reorganization process. This definition can be considered as a natural extension of the storage capacity defined for associative memory model [22]. To this end, we define a binary matrix 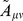 as
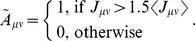



We remove the columns and rows that give vanishing diagonal elements 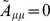 because cell assembly *µ* no longer exists in such a case. We then counted the number of disconnected subgraphs in the graph generated from the resultant adjacency matrix (Fig. 8*C*: in this case the storage capacity is 12), which should be equivalent to the storage capacity. We found that the storage capacity depends on the strength of STD, and vanishes for too strong STD (Figure 8D). Furthermore, whether a particular cell assembly survives or merges into a larger assembly strongly depends on the initial weight matrix (Methods). If some initial cell assemblies have weak intra-assembly connections, they are unlikely to survive (Figure 8E). Two assemblies are likely to merge into a single assembly if one or both directions of the inter-assembly connections are strong (Figure 8F). Thus, when excitatory connections obey STDP and STD, the network has a limited capacity that is maintained by eliminating “weak” assemblies and integrating strongly linked assemblies into single assemblies.

## Discussion

We have shown that interplays between STDP and STD enrich synaptic weight dynamics in recurrent neural networks, and cause critical effects on the cell assembly retention and modulation in the timescales of seconds and minutes. Some cell assemblies merge into a larger assembly or others are eliminated, and the resultant neuronal circuit is able to retain a finite number of memory traces. In these processes, STD crucially influences the stability of modifiable synapses against noisy background activity.

### Implications in cortical memory processing

Our model proposes a possible circuit mechanism for the long-term retention of selective memory traces encoded by external stimuli into subnetworks of highly connected neurons. In a long time scale, molecular and cellular mechanisms are necessary to maintain synaptic memory traces [21], and it is unlikely that constant reactivation of synapses is permanently necessary for retaining memory. Nevertheless, many experimental results indicate the importance of reactivation of memory traces in learning [3], [4]. During wakefulness, sensory experiences cause positive changes in cortical circuits, and the elevated cortical activity may generate corresponding memory traces in the hippocampus [23]. In the present simulations, we mimicked this encoding process by exposing the network model to external stimuli. We showed that the strength of STD has to be kept within an adequate range for embedding the stimuli as multiple cell assemblies in the network.

Our results suggest that these memory traces undergo flexible modifications through the internal network dynamics, and consequently only strong memory traces are preserved in the circuits (Figure 8E). Moreover, if some assemblies are initially linked with stronger excitatory connections, where the initial connection strength is determined by the strength of external stimuli (Figure 2E), the internal dynamics likely integrate these assemblies into one large assembly to co-activate them in the equilibrium network state. These results seem to be consistent with some properties of episodic memory processing by the brain. It is known in humans that sleep enhances the formation of relational memory [24] and false memory [25]. Though our model is too oversimplified to replicate characteristic neural activity during sleep, it explains that initially correlated memory traces can merge together through a repeated reactivation of the corresponding cell assemblies (Figure 8F). Direct experimental evidence supporting this result is awaited.

### Possible implications in memory deficits and cortical development

A recent study shows that mice lacking cbl-b, a cell signaling related gene widely expressed in the hippocampus of rodents, display an improved performance in long-term memory retention tasks. In these mice, paired-pulse facilitation at Schaffer collateral-CA1 synapses is enhanced, but long-term synaptic potentiation shows no difference [13]. Because paired-pulse facilitation is enhanced at low release probabilities [26], our model with weaker STD may account for the enhanced memory retention of cbl-b null mice observed in experiments. Our model may also explain the relationship between the accumulation of amyloid-β and pathological memory dysfunction. Accumulated amyloid-β is known to disturb long-term potentiation in the hippocampus [27] and this disturbance is often considered as the potential mechanism of dysfunction. Our model implies that an enhanced short-term depression, which actually occurs in the presence of an excess amount of amyloid-β [14], may disturb memory retention. It is also known that corticosterone, a hormone controlling stress-induced memory improvement and impairment [28], modifies the probability of presynaptic glutamate releases in the hippocampus of mice [29]. Thus, our model suggests that modifications in short-term plasticity may provide a universal mechanism to control the stability of memory traces in pathological neural circuits.

Our results are possibly relevant to developmental plasticity as well. It is known that in the primary sensory cortex of rodents, glutamatergic synapses show a weakened short-term depression as the animal grows up. The timing of this change typically coincides with the critical period [30], [31] in which the maturation of GABAergic synapses also occurs [32]. A possible explanation of this coincident timing is that the reduction of STD occurs in order to provide more excitatory current, so that the network can keep a balanced state, despite the growth of inhibitory current. As shown in Figure 4B, our model supports this view. Moreover, our model may explain why the strength of STD has to change with successive developmental stages. If STD were strong in immature animals, STDP would not organize any input-dependent structure in cortical circuits: STD may effectively decouple cortical networks from the influence of afferent inputs from thalamocortical pathways until they are well organized.

### Limitations of the model

Although we pursued biologically plausibility in the present modeling, some assumptions of the model remain to be confirmed by experiment. We assumed that LTD of excitatory synapses has a logarithmic weight dependence, implying that synaptic weights only sublinearly influence the LTD of strong synapses. However, the weight dependence for strong synapses is still unknown. We also implicitly assumed that synaptic weights are solely modified by STDP and homeostatic plasticity within 30 minutes to 1 hour from the application of external stimuli and molecular processes for the consolidation of memory trace occur later. However, the actual synaptic mechanism of memory consolidation is more complicated and remains elusive [21]. In fact, cell assemblies could not be permanently stable in the present model with STD and STDP. Therefore, how these cell assemblies may be maintained in a longer time scale remains open for further theoretical studies. In addition, some predictions of the model should be examined by experiment. Synaptic weights displayed large fluctuations in Figure 3D, which has not been observed in previous experiments. The large-amplitude fluctuations were partly due to our choice of a relatively large learning rate and partly due to the inherent nature of the present log-STDP model. Nevertheless, these fluctuations are unlikely to be harmful to the practical function of synapses because the oscillation amplitude of the mean weight change was less than 1% of the mean synaptic weight (Figure 6D).

### Related previous studies

There are a few recurrent network models that consider both STDP and short-term plasticity. Del Giudice and Mattia showed that a recurrent network with short-term depression is able to robustly organize working memory activity by STDP without destabilizing spontaneous activity [33]. Our results are consistent with this result because STD generates a shallow potential well for memory traces (Figure 6B). We have further investigated recurrent circuits embedding multiple cell assemblies, and found that moderate STD is beneficial to the memory retention through interactions. Our model proposes that interplay between STD and STDP is a possible mechanism of selective retention and integration of memory traces in recurrent neural networks. The role of STD was also demonstrated in recurrent neural networks with STDP for the improvement of pattern separation and pattern completion [34].

A recent study suggests STD is not absolutely necessary to achieve reactivation of clustered neurons in spontaneous activity states of cortical networks [35]. However, this model assumes that individual neurons are in an autonomous oscillatory regime. It is also known that STD is critically important to robustly reproduce irregular low-firing rate persistent activity corresponding to a shallow attractor state [36], [37] in which noise-induced transition can occur. Our study further revealed an important function of synaptic efficiency dynamics caused by STD. During the quiet state of an assembly, synaptic efficiencies recover to an original level in the assembly. As a result, at the beginning of the next reactivation, neurons in the assembly show high firing rates to strengthen intra-assembly synaptic weights.

As for the role of STDP in cell assembly formation, many studies exist [38]. While weight-dependent STDP degrades memory retention compared to additive STDP [39], the log-STDP rule (a variant of multiplicative STDP) used in this study improves the stability of learned network structure, reproducing experimentally observed long-tailed unimodal synaptic weight distributions [40]. Log-normal weight distribution can also be reproduced by network effect [41]. A recent theoretical study showed that stable learning is also possible by considering meta-plasticity in addition to the conventional additive STDP [42]. Multiple cell assemblies were created by inducing symmetry breaking through synchronous spikes [43], correlated inputs [44], [45], or synaptic delays caused by topological network structure [46]. Other models made use of additional mechanisms such as oscillatory dynamics [47], voltage-dependence [48], triplet STDP [49], or specific network configurations [50]. In some works short-term plasticity was also introduced [46], [50], though its functional role was not intensively discussed in these studies. The effects of neuromodulation were also considered, in which neuromodulators scaled up the learning speed and scaled down the synaptic weight [49]. Further experiments are required to select these theoretical proposals.

## Methods

### Model configuration

We construct a recurrent circuit model based on the chaotic balance network model [51], [52] and extend it to include both short-term and long-term plasticity. The network consists of *N*_E_ excitatory neurons and *N*_I_ inhibitory neurons (*N*_E_ = 2500, *N*_I_ = 500), connected randomly with connection probability c_XY_ (X,Y = E or I). We defined connection matrix 
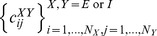
in which 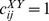 if there is a synaptic connection from *j* to *i*, otherwise 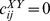. For simplicity, we consider the case where only synaptic connections between excitatory neurons show both types of plasticity, while the weights of excitatory to inhibitory, inhibitory to excitatory, and inhibitory to inhibitory connections are kept at constant values *J_IE_*, *J_EI_*, and *J_II_*, respectively. We use binary neurons taking only two states, 0 or 1. The states of the *i*-th excitatory and inhibitory neurons are defined as 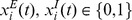. The state of each neuron is updated at time 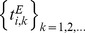 or 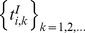 according to a random process with the average intervals *t*_E_^ud^ and *t*_I_^ud^, respectively. This update procedure was asynchronous in the sense that we updated 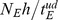 excitatory and 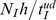 inhibitory neurons at every *h* milliseconds (*h* = 0.01 milliseconds; *t*_E_^ud^, *t*_I_^ud^ = 5.0 and 2.5 milliseconds, respectively). The use of binary neurons and discrete update rule reduces the computational load of the simulation of a large recurrent network model with long-term plasticity. The update rules are written as
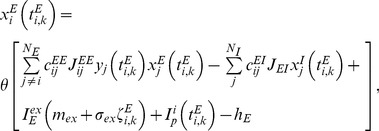
(4)

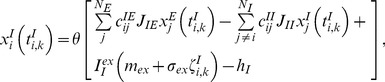
(5)Where θ[*x*] is a step function, and *y_j_(t)* is the synaptic efficiency, representing the effect of short-term depression. The terms *I_E_^ex^m_ex_* and *I_I_^ex^m_ex_* are the fixed components of the amplitudes of random external inputs to excitatory and inhibitory neurons, respectively, while 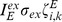 and 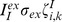 are the random components of those external inputs. The noise terms 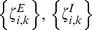 are Gaussian random variables with mean 0 and variance 1. The additional external current 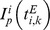 is *I_p_* only for excitatory neurons in the stimulated assembly during the external stimulation, and otherwise remains zero. In the present simulation, we typically applied *I_p_ = 1.0* to 500 selected excitatory neurons for tens of seconds. The variables *h_E_*, *h_I_* are the thresholds of the neurons. Once updated, each neuron keeps its state until the next update. For instance, if 
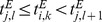
, then 
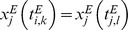
. We did not introduce a reset procedure mimicking a repolarization process after spiking, because inputs to a neuron are refreshed by every update of the neuron. Excitatory neurons stay in the spiking state for 5 msec on average, while inhibitory ones continue to fire typically for 2.5 msec. Thus, neurons rarely stay in the spiking state for a long time due to the randomness of update. Note *J_ij_^EE^* is normalized such that the size of the first EPSP is the same ( = *J_ij_^EE^*) for different release probabilities. This means that the total synaptic weight *J_ij_^EE^_max_* is given as *J_ij_^EE^_max_ =  J_ij_^EE^/u_sd_*. Under this normalization, we can investigate the effect of STD without interference from absolute synaptic weights.

Short-term plasticity is approximately described by the spiking activity of presynaptic neuron [9]. Namely, synaptic efficiency *y_j_* is described with the differential equation
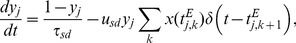
(6)where *u*_sd_ is the release probability and *τ_sd_* is the recovery time constant (*τ_sd_*  = 0.6 second). In numerical simulations, we discretize the time variable such that the synaptic efficiency decreases at the next update when a presynaptic neuron fires.

For long-term plasticity, we consider log-STDP [40] and homeostatic plasticity. Log-STDP is a spike-pair-based STDP-model with a logarithmic weight dependence of LTD (Figure 1A). It was modeled to account for the long-tailed, typically lognormal, distributions of the strength of excitatory synapses in the hippocampus and neocortex [53] [54]. The synaptic weight change for two spikes at t_pre_ and t_post_ is written as
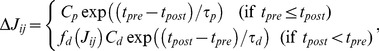
(7)where 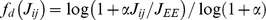 and, *τ_p_* and *τ_d_* are the decay time constants of LTP and LTD respectively (*τ_p_* = 20, *τ_d_* = 40 milliseconds). In calculating the time differences between pre- and post-synaptic firing for STDP, we define the time of firing of a neuron as the time of update at which its state becomes 1. Conduction delays between neurons were not taken into account. If a neuron remains in the spiking state for two consecutive bins, those events are regarded as the generation of two spikes. In addition, we consider the effect of homeostatic synaptic plasticity as
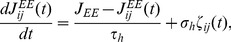
(8)with Gaussian random noise 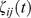. Time constant *τ_h_* of homeostatic plasticity need to be sufficiently short in order to stabilize the network with STDP, while that should be long enough not to erase learned structure rapidly [55]. We set *τ_h_* in order of minutes in the simulation.

Finally, to ensure the stability of the recurrent network, we set boundary conditions for excitatory synapses as 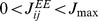 and for the mean excitatory synaptic weight on individual excitatory cells as 
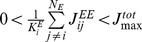
, where *K_i_^E^* is the total number of excitatory inputs to neuron *i*. When the mean excitatory synaptic weight exceeds the upper limit, we subtract the excess amount from all synapses equally.

We used discrete update rule for spiking to reduce the computational cost, and employed differential equations only for slow variables (i.e., synaptic efficacies and homeostatic plasticity). This heterotic update procedure makes simulations faster and more robust in a broad range of parameter values without changing the essential features of network dynamics. However, because the exact spike timing depends on the random update of binary neurons, the update of synapses by STDP undergoes additional noise. This large noise seems reasonable because the *in vitro* synaptic modification by STDP is often highly noisy [7], and is expected to be more noisy *in vivo*. To justify the heterotic update procedure, we performed simulations in a similar network of Poisson neuron model. The details of this model are explained in Text S1 and Figure S1.

We compared the behaviors of excitatory synapses for binary, Poisson, and conductance-based integrate-and-fire (LIF) neuron models (Figure S2). The details of LIF model are explained in Text S1. We applied 500 excitatory inputs and 250 inhibitory inputs to each neuron model, where presynaptic firing rates were high (*r_p_*) at 100 excitatory inputs and low (*r_b_*) at the remaining inputs (Fig. S2A). The rate of inhibitory inputs was *r_I_* = 15.0 Hz. Inhibitory weights were tuned to the weight of excitatory synapses fixed at *J_i_^E^* = 1.15*J_EE_* such that the output neuron fires at 2.0 Hz when *r_p_* = *r_b_* = 2 Hz. Poisson neuron model showed similar membrane dynamics with LIF model (Fig. S2B). The weights of (modifiable) excitatory synapses relative to *J_EE_* showed binary behavior in all models and for different values of *r_b_* and STD strength *u_sd_*: the relative weights converged to large values for the high-rate input when *r_p_* was sufficiently strong (Fig. S2C). Here, we chose different background firing rates in different models to obtain similar magnitudes of potentiation for the strong synapses (*r_b_* = 0.2 Hz for binary, 0.5 Hz for Poisson and 0.8 Hz for LIF), as their behavior depends on *r_b_* (Fig. S2D). We notice that synaptic weights for low-rate inputs tend to be larger in Poisson and LIF models than in binary model, which may be the potential cause of the instability known in the self-organizing process of recurrent networks of LIF neuron [56]. Though we obtained similar results in networks of binary and Poisson neuron models (Fig. S1), we will not investigate networks of LIF models in this study.

### Mean-field (MF) approximation of cell-assembly dynamics

When the firing rate of presynaptic neuron *j* is constant, we find from the fixed point of equation (6) that synaptic efficiency *y_j_* converges to 
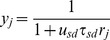
. With this relation, we may use a mean-field approximation for a given synaptic weight configuration [10], [57]. When excitatory neurons are separated into *p* number of non-overlapping cell assemblies with the sparseness *a_1_, a_2_,…, a_p_* (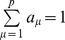), the mean-field equations are calculated as follows:
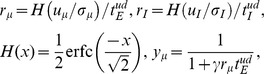


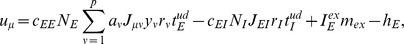


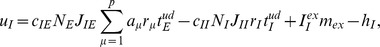


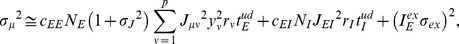


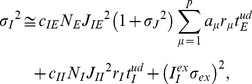
(9)where parameter *σ_J_* is the relative variance of synaptic weight, and *J_µν_* is the average synaptic weight from cell assembly *ν* to *µ*. When the synaptic weight distribution is not Gaussian, as in the case for log-STDP, the mean-field approximation is not accurate unless the correction terms representing the effect of strong synapses are added [58], [59]. However, here we use the above equations for simplicity.

In Figure 6A–C, we calculate the fixed points of equation (9) for two cell assemblies, ca1 and ca2, by substituting *p = 3, a_1_ = 0.2, a_2_ = 0.2, a_3_ = 0.6* (*a_3_* corresponds to the background neurons) to equation (9) and by setting synaptic weights as



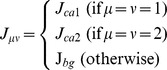
.

In the calculation, we assume that variables *r_I_* and *r_3_*( = *r_bg_*) are slaved to *r_1_*( = *r_ca1_*) and *r_2_*( = *r_ca2_*). As shown in Figure 6E–F, we calculate the average firing rate *r_ca_*(*Δt*) after *Δt* milliseconds of a silent epoch, by substituting the post-silent-epoch efficiency 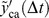 into the corresponding *y_µ_* in equation (9). For instance, in the derivation of *r’_ca_*(*Δt*), we use 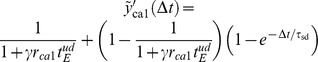 instead of 
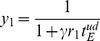
, then calculate the fixed point. Note that we set *r_ca1_* equal to a fixed value estimated from simulations (in Figure 6E, *r_ca1_* = 13.38 [Hz] for *u_sd_* = 0.1 and *r_ca1_* = 10.14 [Hz] for *u_sd_* = 0.2. In Figure 6F, *r_ca1_* = 13.38 [Hz] and *r_ca2_* = 12.82 [Hz]), while *r_1_* is a free variable.

### MF approximation of weight dynamics

We extend the MF approximation to consider the weight dynamics under long-term synaptic plasticity. For simplicity, we assume that the average synaptic weight from a cell assembly to a background neuron pool is the same as the average weight from the background to the cell assembly. In this case, from the MF approximation, the stable point of the network is described by the three parameters *r_I_, r_ca_,* and *r_bg_* corresponding to the average firing rates of inhibitory neurons, excitatory neurons belonging to a cell assembly, and other excitatory neurons (background neurons), and the three parameters *J_ca_, J_m_,* and *J_bg_* representing the average weights of connections inside the cell assembly, between the assembly and the background, and among the background neurons, respectively. Thus, the equilibrium firing rates are expressed as
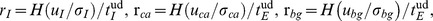


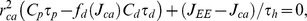


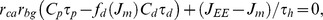


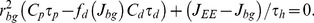
(10)


Note that the above approximation is only applicable under the assumption that the firing rates are uniquely determined for the given synaptic weights. When the firing rates show bi-stability for given synaptic weights, an analytic approach to the synaptic weight dynamics is very hard.

### Initial conditions

We set the initial synaptic weight matrix for simulations as 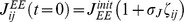 in simulations shown in Figures 2 to 6. Those in Figure 7 and Figure 8A–D, the initial synaptic weight matrix is given as
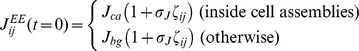
where each cell assembly contains *N_E_a* neurons and 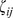 is a Gaussian random variable. Parameter values are chosen as *J_ca_* = 0.70, *J_bg_* = 0.16, *a* = 0.03 and *p* = 32 for the model with a large number of cell assemblies, while *J_ca_* = 0.30, *J_bg_* = 0.16, *a* = 0.2 and *p* = 3 or 5 for the models with a small number of assemblies. In Figure 8E, we introduce an initial bias in the weights within cell assemblies as



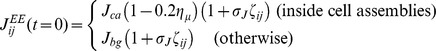
where *η_µ_* is an uniform random variable drawn from 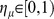 for each cell assembly *µ*. Similarly in Figure 8F, we bias the weights within assemblies as



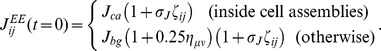



In all simulations, we set other initial conditions as 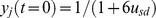, 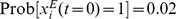, and 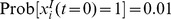.

### Details of simulation

In the presented simulations, every 0.01 milliseconds, 5 excitatory and 2 inhibitory randomly selected neurons are updated. STDP is calculated for neighboring spikes within 500 milliseconds. The differential equations of synaptic efficiency for STD is solved by Runge-Kutta method with 0.1 ms time steps, while homeostatic plasticity is calculated by Runge-Kutta method with 10.0 milliseconds time step in which values are updated at every t = 10.0 milliseconds for t = 0, 10, 20 ms, …. This approximation is reasonable as homeostatic plasticity generates negligibly small changes in synaptic weights at each time step. The parameters used in the present simulations are summarized in Table 1. Code for simulations is written with C++ and Python, and is performed on a cluster machine.

**Table 1:** Parameters used in the simulations.

N_E_, N_I_	Number of excitatory/inhibitory neurons	2500, 500
c_EE_, c_EI_, c_IE_, c_II_	Connection probabilities	0.2, 0.5, 0.2, 0.5
J_IE_, J_EI_, J_II_	Synaptic weights	0.15, 0.15, 0.06 (In Figure 2A and 3, J_EI_ = 0.20)
J_EE_	Standard synaptic weight	0.15
J_EE_^init^, σ_J_	Initial conditions of synaptic weight	0.18, 0.3
I_E_^ex^, I_I_^ex^	Amplitude of steady external input	2.0, 0.5
m_ex_, σ_ex_	Mean and variance of external input	0.3, 0.1
h_E_, h_I_	Thresholds of update	1.0, 1.0
t_E_^ud^, t_I_^ud^	Average intervals of update	5.0, 2.5 milliseconds
H	Interval of state update	0.01 milliseconds
τ_sd_	Decay time constant of STD	600 milliseconds
u_sd_	Release probability of synapse	0.05–0.5
C_p_, C_d_	Coefficients of STDP	0.01875, 0.0075
τ_p_, τ_d_	Decay time constants of STDP	20, 40 milliseconds
α	Degree of log-STDP	50.0
τ_h_	Decay time of homeostatic plasticity	100 seconds
σ_h_	Noise amplitude of homeostatic plasticity	0.00015 per 10 milliseconds
J_max_, J_max_^tot^	Boundary conditions	0.75, 0.25

## Supporting Information

Figure S1 The model with Poisson neuron model. (**A**) Time evolution of the average synaptic weight for three values of *u*_sd_ (*u*_sd_  = 0.15, 0.20, 0.25 from the left side). (**B**) Raster plots of spiking activity corresponding to the three cases shown in **A**. (**C**) Synaptic weight matrices of excitatory connections are shown for the above three cases. Configuration of graphs are the same with Figure 5(**C**), (**D**), (**E**). Details of the model are summarized in Text S1.
(TIFF)

Figure S2Single neuron simulation in different neuron models. (**A**) Schematic illustration of simulation protocol. (**B**) Typical membrane dynamics of Poisson neuron model and LIF model are compared for the same input spike trains. The membrane potential of Poisson model is defined as *v* ^P^°^iss^°^n^ = 10*u*-52 from the dimensionless variable *u*. (**C**) Average relative synaptic weights 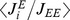 are shown for high- (thick) and low-rate (thin) excitatory inputs to binary (left), Poisson (middle) and LIF (left) neuron models for various values of the release probability *u_sd_*. (**D**) Average relative synaptic weights are shown for high-rate excitatory inputs after 10 minutes of stimulation to binary neuron model.(TIFF)

Text S1 Supporting Materials and Methods.
(PDF)
