# Supplementary figures and images for: Interplay between Short- and Long-Term Plasticity in Cell-Assembly Formation
Source: PLoS One. 2014 Jul 9;9(7):e101535. doi: 10.1371/journal.pone.0101535 (PMC4090127; doi:10.1371/journal.pone.0101535)

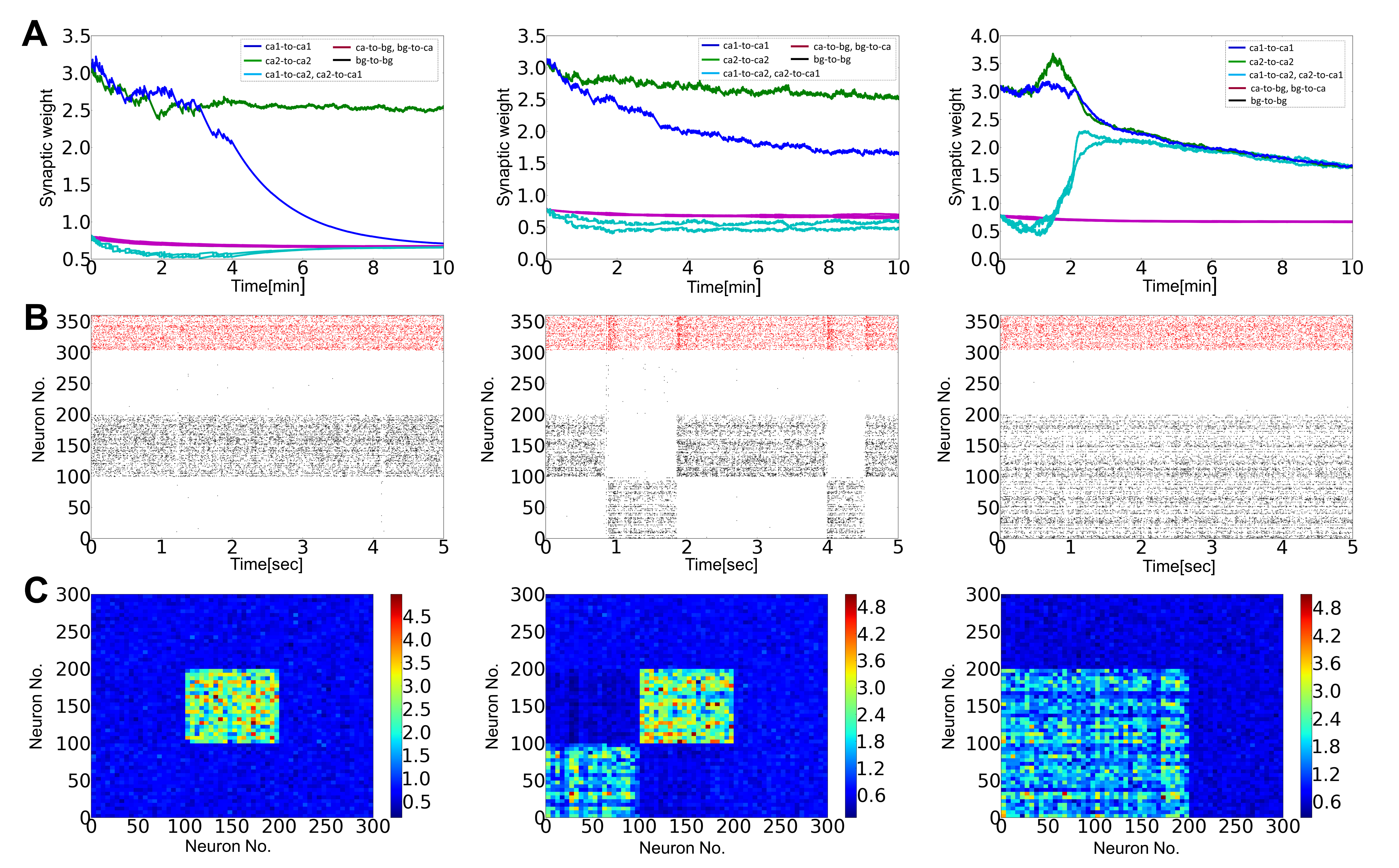

Supplement: Figure S1 — The model with Poisson neuron model. (A) Time evolution of the average synaptic weight for three values of u sd (u sd = 0.15, 0.20, 0.25 from the left side). (B) Raster plots of spiking activity corresponding to the three cases shown in A. (C) Synaptic weight matrices of excitatory connections are shown for the above three cases. Configuration of graphs are the same with Figure 5(C), (D), (E). Details of the model are summarized in Text S1. (TIFF) [file pone.0101535.s001.tiff]

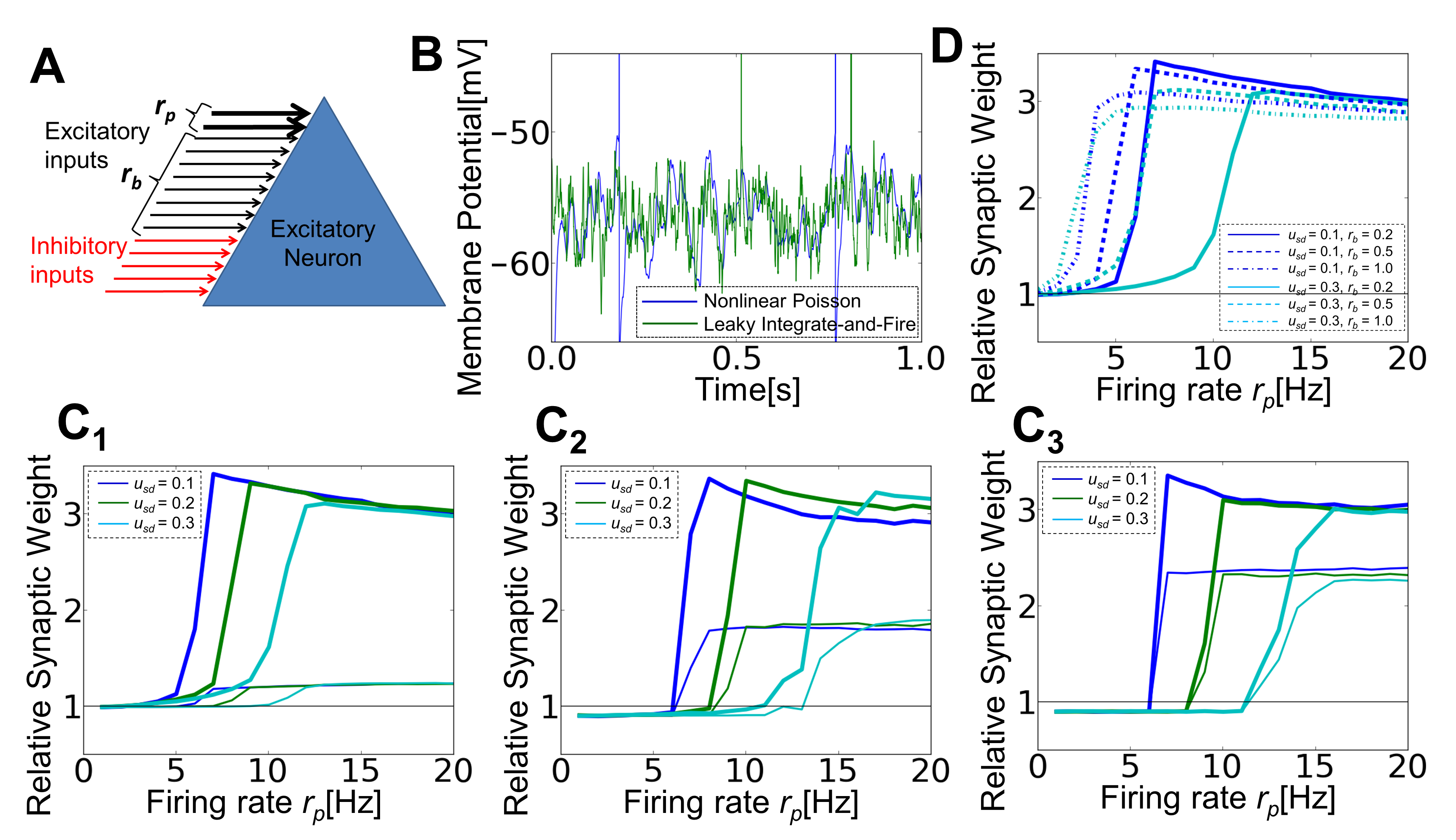

Supplement: Figure S2 — Single neuron simulation in different neuron models. (A) Schematic illustration of simulation protocol. (B) Typical membrane dynamics of Poisson neuron model and LIF model are compared for the same input spike trains. The membrane potential of Poisson model is defined as v P°iss°n = 10u-52 from the dimensionless variable u. (C) Average relative synaptic weights are shown for high- (thick) and low-rate (thin) excitatory inputs to binary (left), Poisson (middle) and LIF (left) neuron models for various values of the release probability usd. (D) Average relative synaptic weights are shown for high-rate excitatory inputs after 10 minutes of stimulation to binary neuron model. (TIFF) [file pone.0101535.s002.tiff]
