# Supplementary material for: Interplay between Short- and Long-Term Plasticity in Cell-Assembly Formation
Source: PLoS One. 2014 Jul 9;9(7):e101535. doi: 10.1371/journal.pone.0101535 (PMC4090127; doi:10.1371/journal.pone.0101535)
Supplement: Text S1 — Supporting Materials and Methods. (PDF) [file pone.0101535.s003.pdf]

Supplemental Text S1

Model with Poisson neuron model

In the main article, we used a binary model for modeling neuron. In order to support the generality of this model, we reproduce the main results of the model with a nonlinear Poisson neuron model [S1,S2].

Excitatory and inhibitory neurons follow spiking dynamics defined as below. Synaptic depression is added only for E-to-E connections.

$$u_i^E(t) = \sum_{j\neq i}^{N_E} J_{ij}^{EE} \int_0^\infty \varepsilon_E(\tau) y_j(t-\tau) x_j^E(t-\tau-d_{ij}^{EE}) d\tau - \sum_j^{N_I} J_{EI} \int_0^\infty \varepsilon_I(\tau) x_j^I(t-\tau-d_{ij}^{EI}) d\tau \tag{1}$$

$$u_i^I(t) = \sum_j^{N_E} J_{IE} \int_0^\infty \varepsilon_E(\tau) x_j^E(t-\tau-d_{ij}^{IE}) d\tau - \sum_{j\neq i}^{N_I} J_{II} \int_0^\infty \varepsilon_I(\tau) x_j^I(t-\tau-d_{ij}^{II}) d\tau \tag{2}$$

$$\varepsilon_E(t) = \frac{\exp(-t/\tau_E^A) - \exp(-t/\tau_E^B)}{\tau_E^A - \tau_E^B}, \quad \varepsilon_I(t) = \frac{\exp(-t/\tau_I^A) - \exp(-t/\tau_I^B)}{\tau_I^A - \tau_I^B} \tag{3}$$

$$\frac{dy_i(t)}{dt} = \frac{1-y_i(t)}{\tau_{sd}} - u_{sd} y_i(t) x_i^E(t) \tag{4}$$

u_i^E, u_i^I are membrane potentials of excitatory/inhibitory neurons calculated by a sum of excitatory and inhibitory currents of a neuron. Synaptic currents are given by convolution of input spikes with EPSP/IPSP curves given as $\varepsilon_E(t), \varepsilon_I(t)$. We assumed that synaptic delays $d_{ij}^{EE}, d_{ij}^{EI}, d_{ij}^{IE}, d_{ij}^{II}$ are uniformly distributed in 0.5-1.5milliseconds for all connections. Synaptic depression is controlled by synaptic efficiency y_i. By membrane dynamics described in equation (1)-(4), spiking process of neurons is given as below.

$$\rho_i^E(t) = \rho_i^{E,ext}(t) + g_E\left(u_i^E(t)\right), \quad g_E(u) = \frac{A_E}{1+\exp(-\lambda u + h_E)} \tag{5}$$

$$\rho_i^I(t) = g_I\left(u_i^I(t)\right), \quad g_I(u) = \frac{A_I}{1+\exp(-\lambda u + h_I)} \tag{6}$$

$$x_i^E(t) \leftarrow Poisson\left(\rho_i^E(t)\right), \quad x_i^I(t) \leftarrow Poisson\left(\rho_i^I(t)\right) \tag{7}$$

Spikes x_i^E, x_i^I are probabilistically generated with sigmoidal response functions $g_E(u)$, $g_I(u)$. We added external inputs $\rho_i^{E,ext}(t) = 10Hz$ to ignite the spiking process at first 100 milliseconds of simulation. After that, external input terms $\rho_i^{E,ext}(t)$ are kept as zero. Synaptic weights of E-to-E connections are modified by STDP and homeostatic plasticity as below.

$$\frac{dJ_{ij}^{EE}}{dt} = x_j^E(t-d_{ij}^{EE}) \int_0^\infty F_d(s, J_{ij}^{EE}) x_i^E(t-s) ds + x_i^E(t) \int_0^\infty F_p(s) x_i^E(t-s-d_{ij}^{EE}) ds + \frac{J_{EE} - J_{ij}^{EE}}{\tau_h} + \sigma_h \zeta \tag{8}$$

$$F_d(s, J_{ij}^{EE}) = C_d\left(1+\sigma_{stdp}\zeta\right) \frac{\log(1+\alpha J_{ij}^{EE} / J_{EE})}{\log(1+\alpha)} \exp(-s/\tau_d), \quad F_p(s) = C_p\left(1+\sigma_{stdp}\zeta\right)\exp(-s/\tau_p) \tag{9}$$

To guarantee stability of the model, we set lower/upper boundaries ($0 < J_{ij}^{EE} < 10 J_o^{EE}$) to E-to-E connections. We chose the same parameter with the model in the main text for time constant of STD, STDP, and homeostatic plasticity. Parameters used in the simulation are summarized in

Supplemental Table S1. All differential equations are solved with Runge-Kutta method with interval h = 0.1milliseconds.

As the simulation tends to take a long time, we created relatively small network with 300 excitatory neurons and 60 inhibitory neurons. Also, because the robustness in parameter space is relatively limited [S3], we simulated only one configuration corresponding to Figure 5, at a given parameter set. We introduced two cell assemblies each consists of 100 non-overlapping excitatory neurons by hands with following equations for a Gaussian random variable ζ_{ij}.

$$J_{ij}^{EE}(t=0)=\begin{cases}4J_{init}^{EE}\left(1+\sigma_J\zeta_{ij}\right) & \text{(inside cell assemblies)}\\ J_{init}^{EE}\left(1+\sigma_J\zeta_{ij}\right) & \text{(otherwise)}\end{cases}$$

Then, observed dynamics change of synaptic weights and neural activity after a dozen minutes of spontaneous activity. As a result, the network showed similar phenomena with those we observed in Figure 5. When STD is weak (i.e., u_{sd} is small), two assemblies show competition, then eventually one of them become dominant (Figure S1-left, $u_{sd}=0.15$). On the other hand, at strong STD, two assemblies tend to merge each other (Figure S1-right, $u_{sd}=0.25$). At the adequate level of STD, both of them survive by alternative excitation (Figure S1-center, $u_{sd}=0.20$).

In order to obtain the results shown for the Poisson neuron model, g_E(u) needs to be a sigmoid-type function. When g_E(u) is linear, bi-stable state is not robustly attained, while g_E(u) is exponential, the network tends to display epileptic states. In addition, synaptic weight changes by STDP need to be noisy. On the other hand, in the original model σ_{stdp} was zero because the model has intrinsic noise due to probabilistic updating.

Conductance-based integrate-and-fire model

In conductance-based integrate-and-fire model we used in Supplementary Figure S2, the membrane potential v follows,

$$\frac{dv}{dt}=-\frac{1}{\tau_m}(v-V_L)-g_E(v-V_E)-g_I(v-V_I)$$

$$\frac{dg_E}{dt}=-\frac{g_E}{\tau_s^E}+\sum_{i=1}^{N_E}J_i^E\sum_{s_i}\delta(t-s_i),\quad \frac{dg_I}{dt}=-\frac{g_I}{\tau_s^I}+\sum_{i=1}^{N_I}J_i^I\sum_{s_i}\delta(t-s_i),$$

where g_E and g_I are excitatory and inhibitory conductance respectively. If $v>V_{th}$, the neuron fires a spike, then the membrane potential goes back to $v=V_L$. Membrane constants were given as $V_L=-70.0$, $V_E=0.0$, $V_I=-80.0$, $V_{th}=-50.0$ millivolts, and time constants were chosen as $\tau_m=20.0$, $\tau_s^E=5.0$, $\tau_s^I=2.5$ milliseconds. Synaptic conductance were set as $J_E=0.004$, and $J_I=\phi_I J_E$. Parameter ϕ_I was adjusted so that the output firing rate becomes 2.0 Hz when excitatory input firing rates are 2.0 Hz and inhibitory rates are 15.0Hz. Short-term plasticity and long-term plasticity were implemented in the same way as we did for nonlinear Poisson neuron model. As shown in Figure S2,

Poisson neuron model and IAF model, and also binary neuron model show similar synaptic weight development for different excitatory firing rates and the strength of STD.

References for Supplemental Text

S1 Gerstner W, Kistler WK (2002) Spiking Neuron Models. Cambridge, UK: Cambridge University Press.
S2 Gilson M, Burkitt AN, Grayden DB, Thomas DA, van Hemmen JL (2009) Emergence of network structure due to spike-timing-dependent plasticity in recurrent neuronal networks I: Input selectivity–strengthening correlated input pathways. Biol Cybern 101: 81-102.
S3 Morrison A, Aertsen A, Diesmann M (2007) Spike-Timing-Dependent Plasticity in Balanced Random Networks. Neural Comput 19: 1437-1467.

Supplemental Table S1

N_E, N_I	Number of excitatory/inhibitory neurons	300, 60
c_{EE}, c_{EI}, c_{IE}, c_{II}	Connection probabilities	0.5,1.0,1.0,1.0
J_{IE}, J_{EI}, J_{II}	Synaptic weights	1.333,0.6J_{EE},0.333
J_{EE}	Standard synaptic weight	0.667
d_{ij}^{EE},d_{ij}^{EI},d_{ij}^{IE}, d_{ij}^{II}	Synaptic delays	0.5-1.5 milliseconds
J_{EE}^{init}, σ_J	Initial conditions of synaptic weight	1.15 J_{EE}, 0.1
A_E ,A_I	Maximal firing rates	100, 200Hz
h_E, h_I	Thresholds of f-I curve	0.5,2.0
λ	Amplitude of f-I curve	70.0
τ_E^A, τ_E^B	EPSP-curve	5.0, 1.0 milliseconds
τ_I^A, τ_I^B	IPSP-curve	2.5, 1.0 milliseconds
τ_{sd}	Decay time constant of STD	600 milliseconds
u_{sd}	Release probability of synapse	0.15-0.25
C_p, C_d	Coefficients of STDP	0.125 J_{EE}, 0.05 J_{EE}
τ_p, τ_d	Decay time constants of STDP	20, 40 milliseconds
α	Degree of log-STDP	50.0
σ_{stdp}	Noise amplitude of STDP	1.0
τ_h	Decay time of homeostatic plasticity	100 seconds
σ_h	Noise amplitude of homeostatic plasticity	0.0001 J_{EE} per 0.1 milliseconds
